# An overview of physico-chemical mechanisms of biogas production by microbial communities: a step towards sustainable waste management

**DOI:** 10.1007/s13205-016-0395-9

**Published:** 2016-02-16

**Authors:** Ramansu Goswami, Pritam Chattopadhyay, Arunima Shome, Sambhu Nath Banerjee, Amit Kumar Chakraborty, Anil K. Mathew, Shibani Chaudhury

**Affiliations:** 1 Department of Environmental Studies, Visva-Bharati, Santiniketan, West Bengal 731 235 India; 2 Department of Zoology, University of Delhi, New Delhi, 110121 India

**Keywords:** Biogas, Anaerobic digestion, Microbial community, Key genes

## Abstract

Biogas is a combination of methane, CO_2_, nitrogen, H_2_S and traces of few other gases. Almost any organic waste can be biologically transformed into biogas and other energy-rich organic compounds through the process of anaerobic digestion (AD) and thus helping in sustainable waste management. Although microbes are involved in each step of AD, knowledge about those microbial consortia is limited due to the lack of phylogenetic and metabolic data of predominantly unculturable microorganisms. However, culture-independent methods like PCR-based ribotyping has been successfully employed to get information about the microbial consortia involved in AD. Microbes identified have been found to belong mainly to the bacterial phyla of *Proteobacteria*, *Chloroflexi*, *Firmicutes* and *Bacteroidetes*. Among the archaeal population, the majority have been found to be methanogens (mainly unculturable), the remaining being thermophilic microbes. Thus, the AD process as a whole could be controlled by regulating the microbial consortia involved in it. Optimization in the feedstock, pH, temperature and other physical parameters would be beneficial for the microbial growth and viability and thus helpful for biogas production in AD. Besides, the biogas production is also dependent upon the activity of several key genes, ion-specific transporters and enzymes, like genes coding for methyl-CoM reductase, formylmethanofuran transferase, formate dehydrogenase present in the microbes. Fishing for these high-efficiency genes will ultimately increase the biogas production and sustain the production plant.

## Introduction

Worldwide energy consumption and demand are continuously growing up. But, most of the resources used like petroleum, natural gas, coal are not sustainable sources of energy. Numbers of countries in the world including India are currently passing through the critical phase of population explosion and the growing population demands more energy inputs. Therefore, the whole world is now concerned about sustainable renewable energy. As a burning example, the European Union policies have set a fixed target of supplying 20 % of the total European energy demands by the year 2020 from renewable energy systems (Holm-Nielsen et al. 2009).

Biogas technology seems promising to attain sustainable energy yields without damaging the environment only when it is produced through anaerobic digestion (AD) and recovered properly (Qiang et al. 2012; Chojnacka et al. 2015). It is composed of 50–75 % methane, 25–50 % carbon dioxide, 0–10 % nitrogen, 0–3 % hydrogen sulfide, 0–1 % hydrogen and traces of other gases. The term “anaerobic” suggests that the process occurs in the absence of free oxygen and produces CH_4_ through decomposition of waste in nature and reduces environmental pollution (Ward et al. 2008; Qiang et al. 2012).

The biogas process comprises of four stages (hydrolysis, acidogenesis, acetogenesis, methanogenesis) which are catalyzed by different and specialized microorganisms. Although AD processes have been carried out for several decades, knowledge about the microbial consortia involved in this process is limited due to lack of phylogenetic and metabolic data of these predominantly uncultivable microorganisms (Sträuber et al. 2012; Wirth et al. 2012; Chojnacka et al. 2015). Due to the wide variety of starting products, a complex array of microbial species are involved in the AD process, including some obligatory syntrophic organisms, which have greatly limited the value of traditional microbiological methods (O’Flahert et al. 2006; Wirth et al. 2012; Chojnacka et al. 2015). The microbial biogas production is solely an enzyme-driven process involving several ion-specific transporters but the functions of the majority of genes involved in various stages of AD are yet to be explored (Narihiro and SeKiguchi 2007; Demirel and Scherer 2008; Weiland 2010).

Though there are several reviews available on different aspects of biogas production there is a dearth of knowledge related to the different microbial community involved in different steps, the different role they play in each step, key genes involved and how to control these microbial communities to get optimal production of biogas (Wirth et al. 2012). In this review we have tried to focus on the core aspect of the biogas production which is the microbial community. We have also discussed on how to control this microbial community and their key genes involved in this process. Our review might also be helpful for the researcher to focus on fishing the important genes involved in this process to develop smarter biogas production plants.

### Sustainable production of biogas through anaerobic digestion (AD)

The biogas is a sustainable source of energy because, (1) it is fully energy self-sufficient (itself produce the heat and electricity to run the process); (2) independent of any fossil fuel; (3) renewable, (4) carbon neutral and (5) reduces the emission of green house gases (GSGs) to the environment. The substrates from plants and animals only emit the carbon dioxide they have accumulated during their life cycle and which they would have emitted also without the energetic utilization. On the whole, electricity produced from biogas generates much less carbon dioxide than conventional energy and thus will be helpful in reducing green house gas emission (http://www.probiopol.de/2_Why_is_biogas_sustainable.41.0.html). Keeping this in mind, at least 25 % of all bioenergy in the future may originate from biogas (Holm-Nielsen et al. 2009). Waste management, manure production, health care and employment generation are the benefits of biogas system.

### Microbes involved in biogas production

Culture-independent methods including polymerase chain reaction (PCR)-based ribotyping, for the identification and characterization of microbial communities involved in biogas production, have met considerable success in recent times (Wirth et al. 2012; Chojnacka et al. 2015). Chouari et al. (2005) detected the constituents of more than 20 bacterial phyla from anaerobic (mostly methanogenic) waste and wastewater sludge using the culture-independent methods, of them, *Proteobacteria*, *Chloroflexi*, *Firmicutes* and *Bacteroidetes* are most prominent (Chouari et al. 2005). Besides that, in a separate study, characterization of anaerobic microbial community related to biogas production has revealed the presence of *Firmicutes*, *Proteobacteria*, *Actonobacteria*, *Bacteroides*, *Acidobacteria*, *Spirochetes*, *Chloroflexi* (Chojnacka et al. 2015). Recently *Ruminococcus flavefaciens*, *Eubacterium cellulosolvens*, *Clostridium cellulosolvens*, *Clostridium cellulovorans*, *Clostridium thermocellum*, *Bacteroides cellulosolvens* and *Acetivibrio cellulolyticus* have also been reported as predominant fermentative bacteria in the cattle dung-fed digesters and actively involved in the AD process (Nagamani and Ramasamy 1999). In addition to these relatively known taxa, phylotypes belonging to a variety of uncultured phyla (known as ‘clone cluster’) have also been detected in sludge (Chouari et al. 2005).

Methanogens are mostly unculturable microorganisms (Wirth et al. 2012; Chojnacka et al. 2015). Earlier studies have reported that the majority of the Archaeal community identified from anaerobic digesters are very similar to already identified methanogens such as *Methanosarcina barkeri*, *Methanosarcina frisius*, *Methanobacterium formicicum* while the remaining are related to thermophilic microbes such as *Crenarchaea* or *Thermoplasma sp.* (Godon et al. 1997). With respect to the uncultured archaeal lineages, archaeal 16S rRNA gene clones affiliated with the candidate taxon ArcI (a clone cluster at the subphylum (or class) level within the archaeal phylum *Euryarchaeota*) has been retrieved in abundance from a mesophilic methanogenic digester decomposing sewage sludge. It has been proposed that ArcI could be an acetate consumer which might play a role in acetoclastic methanogenesis (Chouari et al. 2005). Another unique, uncultured archaeal taxon that is also often found in methanogenic sludge is subphylum C2 of the archaeal phylum *Crenarchaeota*. Moreover, 16 % of the archaeal rRNA gene clones analyzed from a mesophilic methanogenic digester has been found to belong to members of *Crenarchaeota*, particularly the subphylum C2 (Chouari et al. 2005).

### Biochemical mechanisms of biogas production

**Fig. 1:**
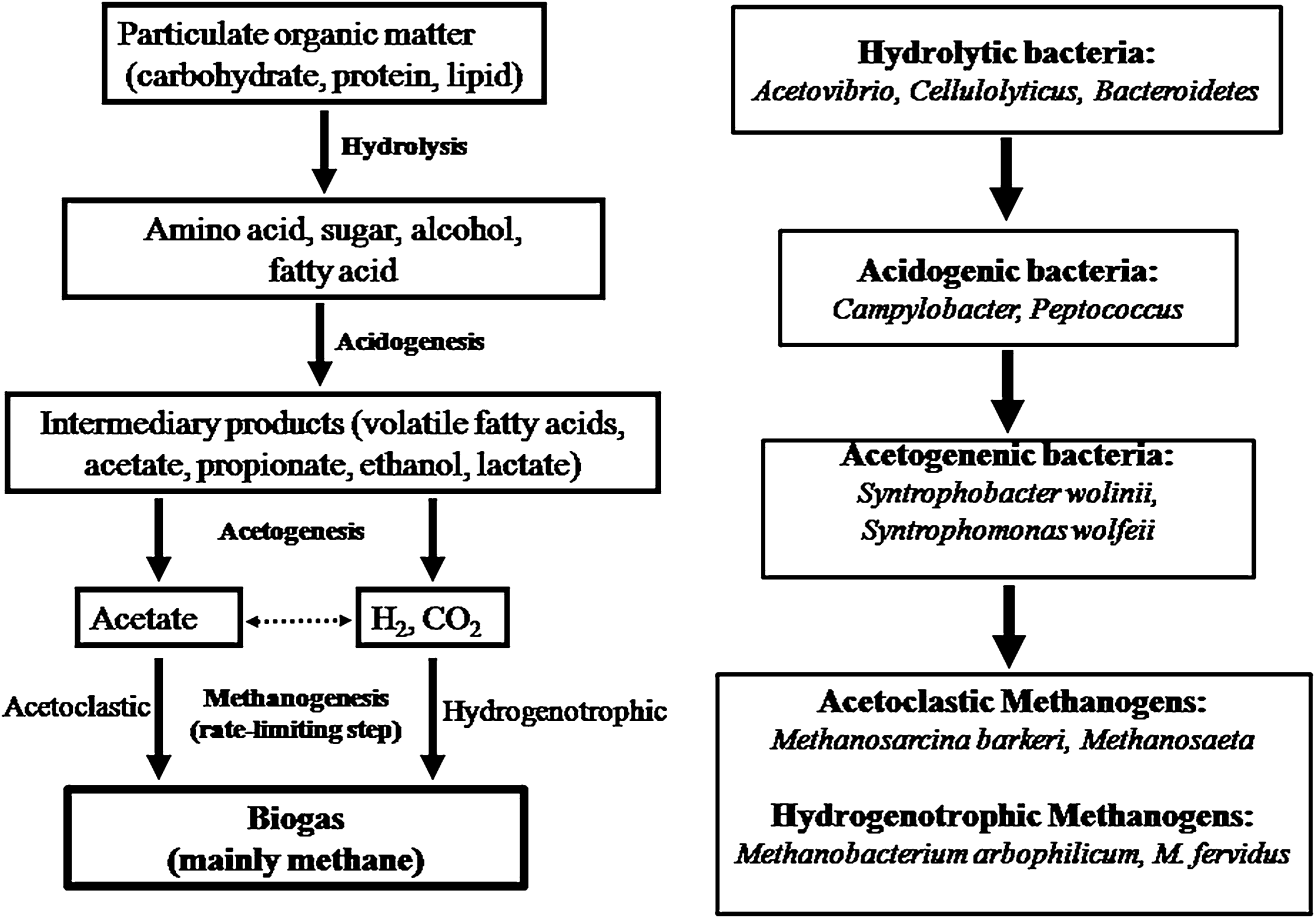
Carbon flow inside the anaerobic digester and bacteria involved in different stages of anaerobic digestion

Biogas production by anaerobic digestion (AD) of wastes is a combinational activity of diverse microbial populations. According to Heeg et al. (2014), the AD chain is initiated by bacteria that are responsible for the hydrolysis of high molecular weight organic substances. Subsequently, the mono- and oligomers produced are further degraded to volatile fatty acids (VFAs) (acidogens) and then to acetic acid, as well as CO_2_ and H_2_ (acetogens). The final step (methanogenesis) is accomplished by acetoclastic and hydrogenotrophic Archaea, which convert acetic acid or CO_2_/H_2_ into methane (Fig. 1).

#### Hydrolysis

The very first step of AD is very important as large organic molecules are not readily absorbable. In this step, several microbes secrete different enzymes, which cleave the complex macromolecules into simpler forms. Organisms that are active in a biogas process during the hydrolysis of polysaccharides include various bacterial groups such as *Bacteriodes*, *Clostridium*, and *Acetivibrio* (Cirne et al. 2007; Doi 2008; Heeg et al. 2014). Some of these organisms have several different enzymes combined into cellulosomes (large, stable, multi-enzyme complexes specialized in the adhesion to and degradation of cellulose that reside with protuberances visible on the cell surface) that are situated on the organism’s cell wall (Liang et al. 2014).

#### Acidogenesis

The diversity of the microbial consortium involved in AD reaches its peak during this stage. Most of the microbes involved in hydrolysis step are also involved in fermentation. Along with them, microbes belonging to the genera like *Enterobacterium*, *Acetobacterium* and *Eubacterium* also carry out the process of fermentation (Schnurer and Jarvis 2010). Through various fermentation reactions, the products from hydrolysis are converted mainly into various organic acids (acetic, propionic acid, butyric acid, succinic acid, lactic acid, etc.), alcohols, ammonia (from amino acids), carbon dioxide and hydrogen. Exactly which compounds are formed depends on the substrate and environmental process conditions, as well as on the microbes present (Schnurer and Jarvis 2010).

#### Acetogenesis

In this step, the fermented products are oxidized into simpler forms. According to Heeg et al. (2014), this step in the AD process requires close co-operation between the microbes that carry out oxidation and the methanogens that are active in the next stage (which actually produce methane). Substrates for acetogenesis consist of various fatty acids, alcohols, some amino acids and aromatics (Heeg et al. 2014). In addition to hydrogen gas, these compounds primarily form acetate and carbon dioxide (Heeg et al. 2014). *Syntrophomonas*, *Syntrophus*, *Clostridium*, and *Syntrobacter* are examples of genera in which there are numerous organisms that can perform acetogenesis in syntrophy with an organism that uses hydrogen gas (McInerney et al. 2008).

### Methanogenesis: the key step for methane production

**Fig. 2:**
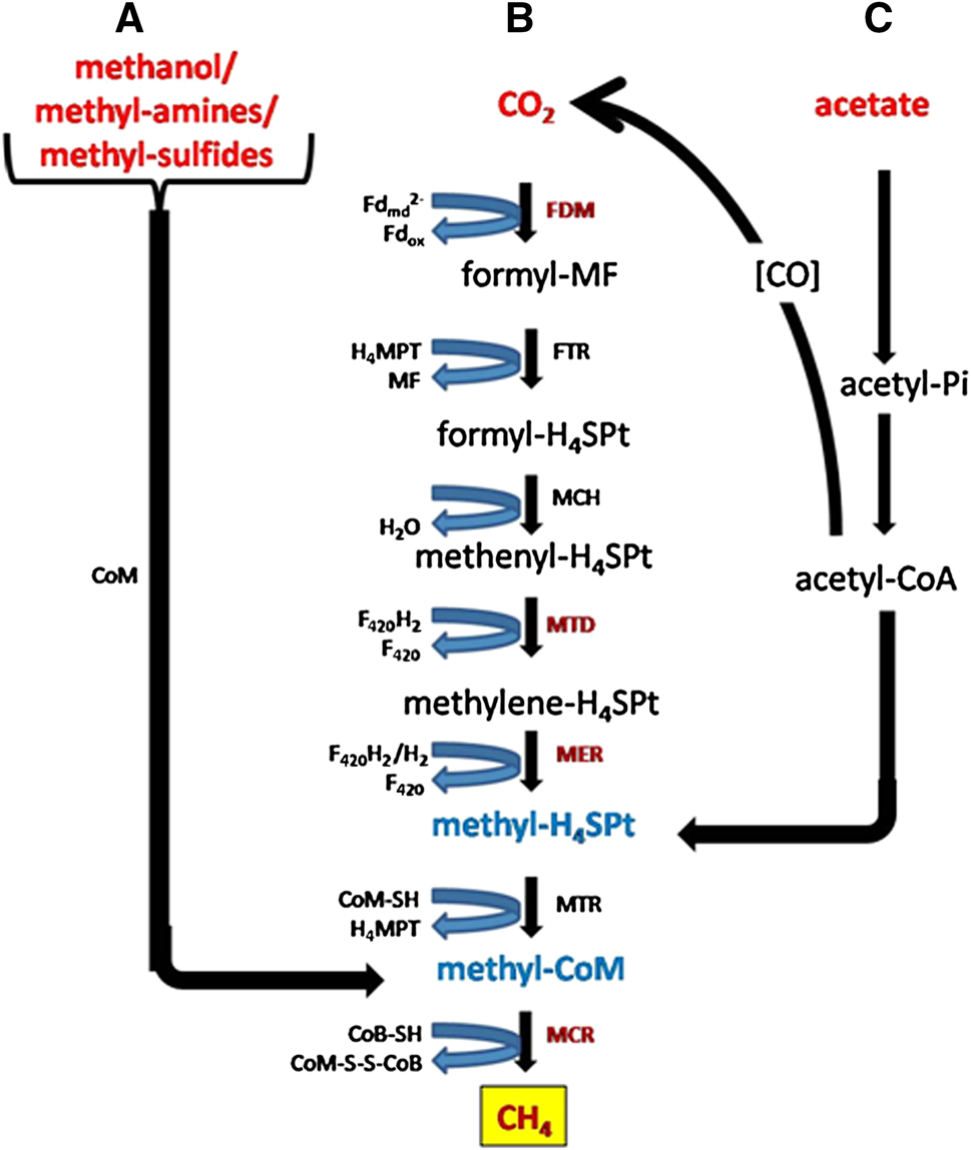
Biochemical pathways to produce CH_4_ from different starting material during AD. **a** Methylotrophic methanogenesis. **b** Hydrogenotrophic methanogenesis. **c** Acetotrophic methanogenesis. MF, methanofuran; CHO-MF, formylmethanofuran; Fd_red_^2−^, reduced ferrodoxin; Fd_ox_, oxidized ferredoxin; FDM (W/Mo-FMD), (tungsten/molybdenum-dependent) formylmethanofuran dehydrogenase; H_4_MPT, tetrahydromethanopterin; FTR, Formylmethanofuran: tetrahydromethanopterin formyltransferase; CHO–H_4_SPT, formylmethanofuran; MCH, *N* ^5^,*N* ^10^-methenyl tetrahydromethanopterin cyclohydrolases; CH≡H_4_SPT^+^, methenyl tetrahydromethanopterin; F_420_H_2_, reduced cofactor F_420_; MTD, coenzyme F_420_-dependent *N* ^5^,*N* ^10^-methylene tetrahydromethanopterin dehydrogenase; CH_2_=H_4_SPT, methylene tetrahydromethanopterin; MER, *N* ^5^,*N* ^10^-methylene tetrahydromethanopterin reductase; CH_3_–H_4_SPT, methyl tetrahydromethanopterin; CoM–SH, coenzyme M; MTR, *N* ^5^-methyl tetrahydromethanopterin: Coenzyme M methyltransferase; CH_3_–S–CoM, methyl coenzyme M; CoB–SH, coenzyme B; MCR, methyl coenzyme M reductase; CoM–S–S–CoB, coenzyme M-HTP heterodisulfide

Methanogenesis (final step inside AD) is the methane production pathway which methanogens follow to obtain energy (Fig. 2). This process involves the fermentation of various organic compounds with methane gas as the major end product along with carbon dioxide, hydrogen and traces of other gases. Methanogenesis has six major pathways, each converting a different substrate into methane gas. The six major substrates used are carbon dioxide, formic acid, acetic acid, methanol, methylamine, and dimethyl sulfate (Slonczewski and Foster 2014). The most common pathway converts carbon dioxide into methane through the reduction of H_2_/CO_2_ (Slonczewski and Foster 2014) (Fig. 2). The other five pathways may be converged into two according to various methanogen specific-cofactors. The pathway which leads to the methane production solely depends on the methanogenic consortia and the availability of the suitable substrates that favors the digestion process. Methane is, therefore, a by-product of this anaerobic decomposition process that aims to break down organic acids and produce energy for the microbes present in the environment (Wang et al. 2011). Therefore, the main three pathways (Fig. 2) are:

1. Methylotrophic methanogenesis, i.e., production of methane by decarboxylation of methyl alcohols/methyl amines/methyl sulfides, etc. (Fig. 2a),
2. hydrogenophilic or hydrogenotrophic methanogenesis, i.e., production of methane by the reduction of H_2_/CO_2_ (Fig. 2b); and,
3. acetoclastic or acetotrophic methanogenesis, i.e., production of methane by acetate decarboxylation (Fig. 2c).

It has been reported that acetoclastic methanogenesis is the major pathway of methane production in anaerobic digestion as 70 % of the total methane generated during AD of domestic sewage is via this pathway (Lettinga 1995; Merlino et al. 2013).

Although the role of methanogens and methane production have been extensively studied, the exact process and pathway of methanogenesis is not well described in most literature (Weiland 2010; Wirth et al. 2012; Chojnacka et al. 2015). It is often simply described as the conversion of organic acids or carbon dioxide into methane (Toprak 1995; Wu et al. 2009). The true process of methanogenesis is much more complex and requires specific substrates and cofactors to occur. The two most common methanogenesis substrates are carbon dioxide and acetate. In the carbon dioxide pathway (hydrogenotrophic methanogenesis), CO_2_ is converted into methane and water via the passing of carbon down a series of cofactors. Carbon dioxide is fixed using hydrogen into methanofuran. The carbon is then passed down three cofactors, Tetrahydromethanopterin (H_4_MPT), Coenzyme F420 or 8-hydroxy-5-deazaflavin (F_420_), and 2-Mercaptoethanesulfonic Acid or coenzyme M (HS-CoM), until the carbon reaches coenzyme B (HS-CoB), which serves as the terminal electron acceptor (Slonczewski and Foster 2014). This process depends on the concentration of hydrogen ions as well as the sodium potential to donate electrons from CO_2_ and drive the ATP synthase that ultimately produces energy for the methanogens (Slonczewski and Foster 2014). Due to this, methanogens, unlike many other microbes, require sodium ions for growth. Methanogenesis from acetate (acetoclastic methanogenesis) requires the coupling of H_2_ concentration and a sodium potential to occur and uses the cofactors HS-CoM (coenzyme M) and HS-CoB (coenzyme B) to produce methane (Slonczewski and Foster 2014). Unlike hydrogenotrophic methanogenesis, which produces water as a waste product, acetate methanogenesis produces a molecule called coenzyme M-HTP heterodisulfide (CoM–S–S–CoB), which is a converged form of the two carbons initially entered into the system (Slonczewski and Foster 2014).

### Factors affecting microbial community in AD for biogas production

The anaerobic digestion of organic material is a complex process, involving a number of different degradation steps performed by different members of the microbial consortia. Thus, a number of factors affect the microbial growth which in turn affects the process of anaerobic digestion and hence, the biogas yields (Mathew et al. 2014). As the hydrolytic/acidogenic bacteria and methanogenic Archaea differ widely in their preferred ambience, such as pH optima and nutrient requirements, the success of any process optimization effort crucially depends on the degree to which the growth, metabolism of all microorganisms involved is supported (Heeg et al. 2014). The effects of few such factors have been discussed below:

#### Temperature

Anaerobic digestion is applied under three different temperature ranges, i.e., the mesophilic (25–40 °C), the thermophilic (45–60 °C) and the psychrophilic (<20 °C) (Khalid et al. 2011; Mathew et al. 2014). The structures of the active microbial communities at the two temperature optima are quite different. A change from mesophilic to thermophilic temperature (or vice versa) can result in a sharp decrease in biogas production until the necessary populations have increased in number (Chae et al. 2008).

#### pH

pH plays a major part in anaerobic biodegradation by influencing the activity of the hydrolytic enzymes (Mathew et al. 2014). It has been reported that methanogenesis in an anaerobic digester occurs efficiently at pH 6.5–8.2, while hydrolysis and acidogenesis occurs at pH 5.5 and 6.5, respectively (Lee et al. 2009).

#### C/N ratio

The C/N ratio in the organic material plays a crucial role in anaerobic digestion (Mathew et al. 2014). The unbalanced nutrients are regarded as an important factor limiting anaerobic digestion of organic wastes. It has been reported that the optimal C/N ratio for anaerobic degradation of organic waste is 20–35 (Lee et al. 2009; Mathew et al. 2014). However, in reality, C/N ratios of the feedstocks are often much lower or higher than this (Zhang et al. 2008). Hence, co-digestion of feedstock is employed to improve the C/N ratio.

#### COD

Chemical Oxygen Demand (COD) content describes the amount of oxygen needed to completely oxidize the waste under aerobic conditions, and is determined experimentally by measuring the amount of a chemical oxidizing agent needed to fully oxidize a sample of the waste. It is used as a measure of the oxygen equivalent of the organic matter content of a sample that is susceptible to oxidation by a strong chemical oxidant. Oxygen is not consumed in anaerobic digestion, and so, no reduction of COD can occur. In this situation, COD is removed by converting organic compounds to methane (CH_4_), a significant amount of CO_2_, H_2_ and negligible amounts of other gases like H_2_S (Manariotis et al. 2010; Mathew et al. 2014). So the methane potential of a waste (by microorganisms) is related to the concentration of organics (COD) in it and in the efficiency of the system.

#### Nutritional requirements

The nutrient requirement is a major concern for the stable operation of methane fermentation processes (Mathew et al. 2014). The growth of methanogens is dependent on many ions such as sodium, nickel, cobalt, iron, zinc, magnesium, calcium and potassium cations and molybdate or tungstate and phosphate anions. Except sodium, which is required for coupling methanogenesis with ADP phosphorylation, all the other ions are required for the synthesis of enzymes, prosthetic groups, and coenzymes (Hattori et al. 2009; Kaster et al. 2011). It has also been reported that the optimal requirements for Fe, Co, and Ni were identified as 200, 6.0, and 5.7 mg/kg COD removed for the methane fermentation of food waste (Qiang et al. 2012). Methanogenic cell concentrations in excess of 1.32, 1.13, 0.12, 4.8, and 30 g l^−1^ have been found to be limited by Fe at a concentration of 5 mg l^−1^, Zn at 1 mg l^−1^, Cu at 0.1 mg l^−1^, Ni at 1.2 mg l^−1^, and Co at 4.8 mg l^−1^, respectively (Zhang et al. 2008).

### Crucial role of important ion channels in the growth of methanogenic microorganisms

Methanogenesis is one of the most metal enriched enzymatic pathways in biology. Depending on the pathway, exact metal requirements may differ, but the general trends always remain the same. Iron (Fe) is the most abundantly required metal, followed by nickel (Ni) and cobalt (Co), and trace amounts of molybdenum (and/or tungsten) and zinc (Zn). Fe remains as Fe–S clusters used for transport of electrons (Glass and Orphan 2012). Ni is either bound to Fe–S clusters or in the center of a porphyrin unique to methanogens, cofactor F_430_. Cobalt is present in cobamides involved in methyl group transfer; whereas Zn occurs as a single structural atom in several enzymes. Molybdenum (Mo) or tungsten (W) is attached to a ‘pterin’ cofactor to form “molybdopterin” or “tungstopterin”, respectively, and involved in catalyzing two electron redox reactions. Other alkali metals and metalloids, such as sodium (Na) and selenium (Se), are also essential for methanogenesis (David and Alm 2010; Dupont et al. 2006, 2010; Glass and Orphan 2012). All these ions, all of which are required for the synthesis of enzymes, prosthetic groups, and coenzymes, must be taken up from the growth medium.

#### Sodium channel

Sodium ions (Na^+^) are required for coupling methanogenesis with ADP phosphorylation. It is transported by four membrane-bound enzyme complexes, *N* ^5^Methyl-H_4_MPT:CoM methyltransferase (MTR), energy-converting [Ni–Fe]-hydrogenase complexes EHA and EHB, A_1_A_0_ ATP synthase complex AHA, and a sodium ion/proton antiporter NHA (Lang et al. 2015; Kaster et al. 2011).

The methyltransferase enzyme is a four membrane-associated integral membrane-bound complex which requires sodium ions for activity and, in addition to methyl transfer, functions to generate a sodium ion gradient across the membrane. The ATP synthase shows a conserved Na^+^-binding motif, and utilized four sodium ions for the phosphorylation of one ADP (Kaster et al. 2011). Reduction of ferredoxin with H2 via Eha or Ehb was driven by the sodium ion-motive force with a Na^+^ to *e* ^−^ stoichiometry of 1; however, this has not yet been established (Lang et al. 2015). The sodium/proton antiporter is most likely there for pH homeostasis (Kaster et al. 2011).

#### Nickel channel

Nickel ions (Ni^2+^) are required for the synthesis of the [Ni–Fe]-hydrogenase complexes (EhaA-T, EhbA-Q, FrhABG, and MvhADG). EHA and EHB is responsible for catalyzing the reduction of ferredoxin with H_2_ driven by proton-motive force; whereas, FRH catalyzes the reversible reduction of coenzyme F_420_ with H_2_ (Zhang et al. 2009; Kaster et al. 2011). They are also required for the synthesis of the two methyl-CoM reductases: McrABG and MrtABG and the carbon monoxide-acetyl-CoA synthase/decarbonylase complex involved in autotrophic CO_2_ fixation. Although the Ni^2+^ transporter is yet to be identified, one of the two Co^2+^ transporters predicted in two *Methanobacter* species has been proposed to be a Ni^2+^ transporter (Kaster et al. 2011).

#### Cobalt channel

Cobalt ions (Co^2+^) are required for the synthesis of cobalamin in the MTR enzyme complex (containing two cobamide cofactors and eight Fe atoms) and of coenzyme B_12_ in the adenosyl cobalamin-dependent ribonucleotide reductase. They are most probably taken up by the transporter CBIMNOQ (Zhang et al. 2009; Kaster et al. 2011; Glass and Orphan 2012).

#### Iron channel

The iron requirement for methanogenesis is vast; almost every metalloenzyme involved in the methanogenesis pathway contains multiple Fe_2_S_2_, Fe_3_S_4_, or Fe_4_S_4_ clusters (Rao et al. 2011). Ferrous ions (Fe^2+^) are required for the synthesis of iron–sulfur clusters in the [Ni–Fe] hydrogenases, formylmethanofuran dehydrogenases (W/Mo-FMD), heterodisulfide reductase (HDR), ferredoxins (Fd), and [Fe] hydrogenase (HMD) (Kaster et al. 2011). The Fe^2+^ ions are transported by the FeoAB transporter encoded by *feoAB* gene (Rao et al. 2011).

#### Zinc channel

Zinc ions (Zn^2+^) are required for the synthesis of the subunit B of HDR enzyme (involved in CO_2_ reduction with H_2_ to methane) and RNA polymerases. The Zn^2+^ ions are translocated by the high-efficiency ZnuABC/ZupT transporters in *Methanothermobacter marburgensis* and *M. thermautotrophicus* which are regulated by the nickel-responsive transcriptional regulator NikR homolog (Wang et al. 2009; Kaster et al. 2011). However, NikR from *E. coli* can also bind zinc ions, but without having any conformational change in the transporter (Leitch et al. 2007; Kaster et al. 2011).

#### Magnesium channel

The synthetase and kinase enzymes generally use complexes of ATP and ADP with Mg^2+^ as substrates and products. Mg^2+^ is predicted to be taken up by the MgtE system (Rao et al. 2011).

#### Calcium channel

Calcium ions (Ca^2+^) are required for the synthesis of Mch enzyme and a membrane-bound Ca^2+^ ATPase (Qiang et al. 2012; Zhang et al. 2008). It is reported that methane formation in cell suspensions of microorganisms is stimulated by the gradient of Ca^2+^ ions which is driven by the membrane-associated Ca^2+^ ATPase (Kaster et al. 2011). Though the presence of Ni^2+^ and Co^2+^ in the microbial growth media has been reported to antagonize the Ca^2+^ transport, available evidence indicates that if a Ca^2+^ uptake system is present, it must be a high-affinity uptake system (Kaster et al. 2011).

#### Potassium channel

Potassium ions are not directly involved in methanogenesis from CO_2_ and H_2_O, but most of the methanogenic enzymes function optimally only at high concentration of K^+^ ions. Most methanogenic bacteria have developed K^+^ transporters and channels, which have enabled them to withstand different environmental stresses. Basically, K^+^ channels are ion-selective pores, composed of two or four subunits, which conduct selective uptake of potassium ions along the electrochemical gradient. The potassium ions are most probably taken up by the low-affinity TrkAH system (Zhang et al. 2009; Kaster et al. 2011).

#### Molybdate/tungstate channel

Molybdate ions (MoO_4_^2−^) are required for the synthesis of the Mo-formylmethanofuran dehydrogenase (MO-FMD), formate dehydrogenase, and nitrogenase and are most likely taken up by the ABC transporter ModA1B1C1 (Zhang et al. 2008). Tungstate ions (WO_4_^2−^) are required for the synthesis of the W-FMD and are most likely taken up by the ABC transporter ModA2B2C2 (Zhang et al. 2008; Kaster et al. 2011).

#### Phosphate channel

In methane production from CO_2_ and H_2,_ phosphate ions are required in ATP formation via the A_1_A_0_ ATP synthase and for the synthesis of the coenzyme H_4_MPT, coenzyme B, and the FeGP-cofactor, which contain covalently bound phosphate. The phosphate ions are probably taken up by a PstABCS/PhoU system (Aguena and Spira 2009; Kaster et al. 2011).

### Key genes involved in biogas production

Microbial biogas (methane) production is a genetically regulated process (Fig. 2). The key genes involved in this process are discussed below:

Formylmethanofuran transferase (FTR) catalyzes the transfer of a formyl group from formylmethanofuran (MFR) to tetrahydromethanopterin (H_4_MPT) (Fig. 2). The FTR-encoding gene from *Methanobacterium thermoautotrophicum* has been cloned, sequenced, and functionally expressed in *E. coli.* Formate dehydrogenase (FDH) may sometimes account for 2–3 % of the total soluble proteins in methanogenic cultures (Darcy et al. 1995). The two genes encoding the a± and a^2^ subunits of FDH have been cloned and sequenced from *Methanobacterium formicicum.* In addition, the genes encoding F_420_-reducing hydrogenase, ferredoxin, and ATPase have also been cloned (Darcy et al. 1995; White and Ferry 1992).

Methyl-CoM reductase (MCR) constitutes approximately 10 % of the total protein in methanogenic cultures (Klein et al. 1988). The importance and abundance of MCR inevitably focused initial attention on elucidating its structure and the mechanisms directing its synthesis and regulation. MCR-encoding genes have been cloned and sequenced from *Methanococcus vanielli*, *Methanococcus voltae*, *M. barkeri*, *Methanobacterium thermoautotrophicum* and *Methanothermus fervidus* (Cram et al. 1987; Lehmacher and Klenk 1994).

A considerable amount of information relevant to natural DNA transformations of prokaryotic bacteria has been reported, and the natural competence of methanogens has been elucidated. *Methanobacterium thermoautotrophicum* was transformed by DNA from fluorouracil-resistant strains, resulting in the production of drug-resistant strains. In *Methanococcus voltae*, auxotrophic mutants requiring histidine or purine were reverted with wild-type DNA, although the genetic transformation frequencies were very low (Micheletti et al. 1991). However, Gernhardt et al. (1990) recently made a breakthrough with integration of a vector into *Methanococcus voltae.* Integration vector transformation techniques have been well exploited in yeasts, but not in methanogens. The *his*A gene cloned from the methanogen was used as an integration site in homologous recombination. In methanococcus, a puromycin-resistant gene from *Streptomyces alboniger* was clearly shown to be expressed and stably maintained only under specific pressure conditions (Sandbeck and Leigh 1991). Further characterization of the integration mode revealed that the integration vector was tandemly repeated in chromosomal genes of *Methanococcus maripaludis* under intensive antibiotic pressure (Sandbeck and Leigh 1991). Furthermore, genomic DNA from the recipient methanogen could directly transform *E. coli* to ampicillin resistance, indicating that integrated plasmid vectors can be used as recoverable shuttle vectors between methanogens and *E. coli* (Sandbeck and Leigh 1991).

### Developments in bioreactor technology for sustainable production of biogas

A bioreactor may refer to any manufactured or engineered device or system that supports a biologically active environment (Wu et al. 2009). The process of biogas production takes place in anaerobic conditions and in different temperature diapasons. There are psychrophilic (temperature diapason 10–25 °C), mesophilic (25–40 °C) and thermophilic (50–55 °C) regimes of bioconversion. Biogas production in a thermophilic regime is much higher than for the mesophilic and psychrophilic regimes. Modern thermophilic bioreactors can produce 2–6 m^3^ per m^3^ of installation, which amounts to 5–15 kg of waste on a dry mass base (or 50–150 kg of wet mass). For mesophilic biogas installations, these values are 0.2–0.4 m^3^ per m^3^ of installation and 0.5–1 kg on a dry mass base (or 5–10 kg of wet mass). Biogas reactors, working in a thermophilic regime, can be introduced in agricultural farms where the number of livestock exceeds 5. Biogas produced on such farms can be used not only for cooking and heating water, but for dairy production as well (Wu et al. 2009). Process imbalances and overloading are often accompanied by an accumulation of propionic acid (Marchaim and Krause 1993; Wang et al. 2006). It is generally accepted that the propionic acid concentration should be kept below 1.5 g l^−1^ for proper process operation (Ma et al. 2007), and the ratio of propionic/acetic acid was suggested to be a sufficient indicator of a digester failure (Marchaim and Krause 1993).

**Fig. 3:**
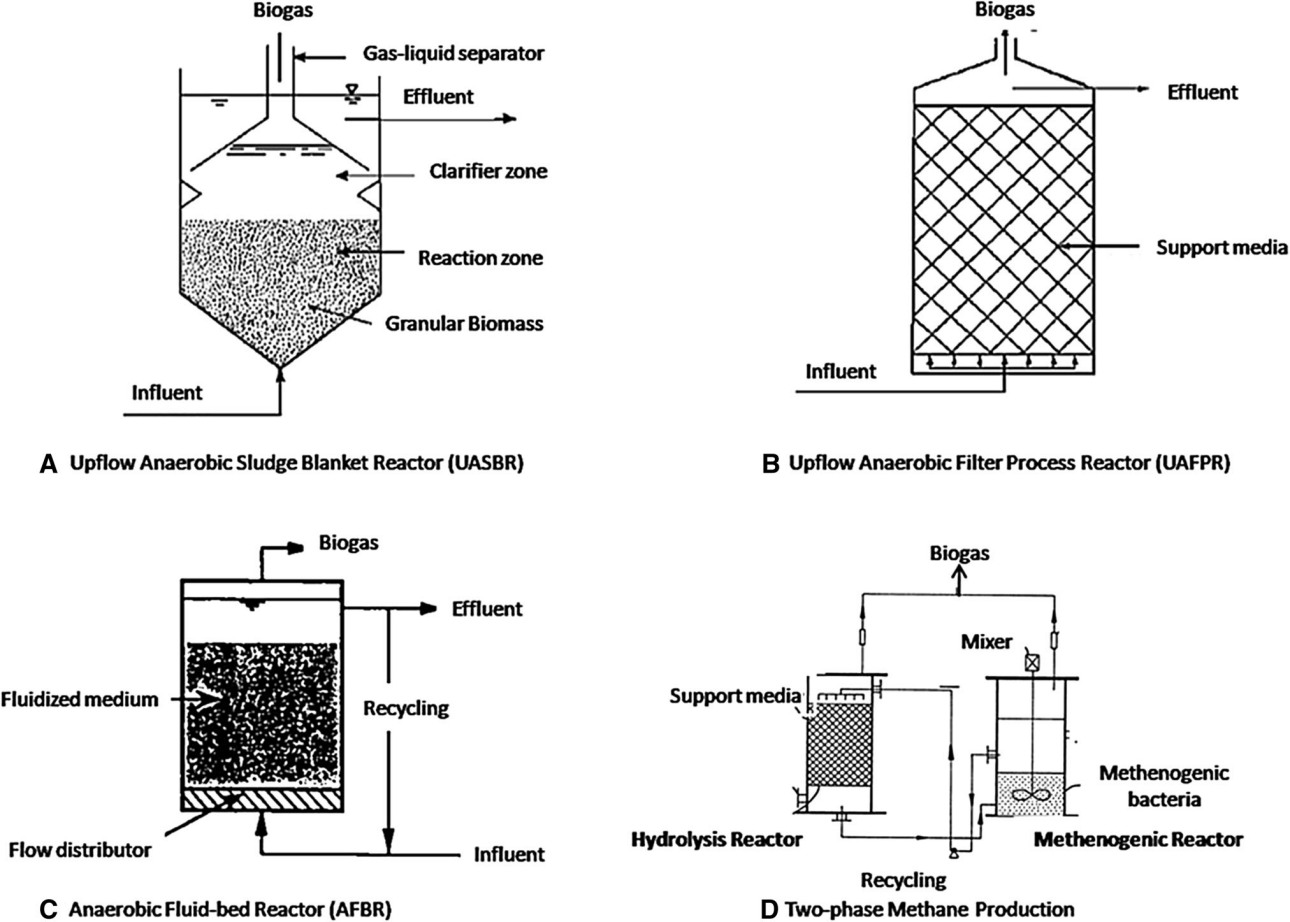
Schematic representation of different bioreactors

For biogas production, research and developmental efforts have been directed at retaining a high density of useful microorganisms, to achieve rapid and effective treatment, with the objective of improving the conventional system. To this end, considerable technological developments in microbial floe formation and in microbial adhesion onto carrier materials which retain cells in the reactor have been made. For the former purpose, the upflow anaerobic sludge blanket (UASB) (Lettinga et al. 1980) has proven useful, while for the latter, the upflow anaerobic filter process (UAFP) (Young and McCarty 1969; Rajathi 2013) and anaerobic fluidized-bed reactor (AFBR) (Jeris 1983; Buffieare et al. 2000) have been developed (Fig. 3). In all of these newly developed processes, however, acidogenesis may occur more frequently than methanogenesis, leading to the accumulation of inhibitory products such as volatile fatty acids. Two-phase anaerobic digestion processes have been developed to resolve this problem (Bowker 1983; Sharma et al. 2012) (Fig. 3).

#### Upflow anaerobic sludge blanket (UASB)

Successful construction of a UASB process capable of affording self-granulation (flocculation) of anaerobic microbes was first reported by Lettinga et al. (1980). In this type of bioreactor, water containing organic waste entering from the bottom of the reactor passes through a sludge bed and sludge blanket where organic materials are anaerobically decomposed. Gas produced is then separated by a gas–liquid separator and the clarified liquid is discharged over a weir, while the granular sludge naturally settles at the bottom (Krzysztof and Frac 2012) (Fig. 3a). Bench- and pilot plant-scale experiments indicate that it is possible to operate this system at a COD loading of 40 kg/m^3^/day at HRTs of 4–24 h (Krzysztof and Frac 2012). Full-scale UASB reactors are now operational in Europe, the US and Japan, with more than 100 recently constructed plants in Japan.

Significant parameters in the UASB operation are floe diameter, microbial density, and the structure of the gas–solid separator which effectively retains the microbial granules within the reactor. The following criteria should be observed to achieve successful UASB operation: (a) selection of a suitable waste water capable of granule self-formation; (b) operation of the reactor without mechanical agitation; (c) start up at a relatively low COD load; (d) use of waste water containing Ca^2+^ and Ba^2+^ and (e) avoidance of bulking caused by filamentous microbial growth. Granule formation in a UASB system is influenced by the growth of rod-type *Methanothrix* spp. which produces spherical granules (Krzysztof and Frac 2012).

#### Upflow anaerobic filter process (UAFP)

UAFP systems were initially developed by Young and McCarty (1969) using rocks and plastics for microbial fixation. These UAFP systems were applied to biogas production from domestic sewage and industrial waste waters containing relatively low levels of organic materials. This type of bioreactor contains a “medium”, i.e., a microbial support (Fig. 3b). Granulated microorganisms exist not only in the spaces within the medium, but are also attached to its surface; hence, a high-density microbial population is retained within the reactor, creating a hybridization of microbial floe and adhesion. To avoid short-circuiting flow through the packed column, a distributor is fitted at the bottom to provide a homogeneous upflow of waste water. At the top, treated waste water and the biogas produced are separated by a free board. Data on full-scale UAFP systems show that alcohol distillery waste water can be treated at an HRT of 7.8 days with 74 % COD removal. Application of this UAFP to domestic sewage treatment using Raschig rings (2.5 cm) as microbial supports, resulted in BOD removal of 50–60 % and suspended solids (SS) removal of 70–80 %, at an HRT ranging from 5 to 33 h (Young and McCarty 1969).

Selection of a medium in which microbial adhesion is greatly influenced both by SS, and the chemical composition of the waste water, is extremely critical in UAFP systems (Mumme et al. 2010). Entrapment of methane-producing microorganisms between semi-permeable synthetic membranes in a multi-layer membrane bioreactor (MMBR) was studied and compared to the digestion capacity of a free-cell digester, using a hydraulic retention time of 1 day and organic loading rates (OLR) of 3.08, 6.16, and 8.16 g COD/L day (Youngsukkasem et al. 2013). The effects of physical medium characteristics, such as size and shape, on COD removal have been investigated using modular corrugated blocks (porosity > 95 %), pall rings, and perforated spheres. At a COD load of 2 kg/m/day, modular corrugated blocks exhibited superior behavior, removing 88 % of COD. A comparison of COD removal for cross- and tubular-flow systems reveals that COD removal is 20–30 % greater in cross-flow systems. In addition to plastic media, baked clay and a melted slug have also proven useful in laboratory experiments on methanogenesis from formate, acetate, and methanol. Pumice was used as a microbial supporter for methanogenesis from methanol-rich waste water of the evaporate condensate from a pulp mill (COD load: 12 kg/m^3^/day, COD removal: 96 %) (Youngsukkasem et al. 2013).

#### Anaerobic fluidized-bed reactor (AFBR)

In this type of systems, the medium to which the microbes adhere is fluidized within the reactor, resulting in conversion of organic materials to CH_4_ and CO_2_ (Krzysztof and Frac 2012) (Fig. 3c). Anaerobic microbes grow on the surface of the medium, expanding the apparent volume of the medium; hence this reactor is also designated an “expanded bed reactor”. Use of artificial sewage in an AFBR, resulted in COD removal exceeding 80 % at 20 °C, and at a COD load of 2–4 kg/m^3^/day this system was tolerant of shock loading for step changes of temperature from 13 to 35 °C and from 35 to 13 °C. In the case of COD shock loading from 1.3 to 24 kg/m^3^/day, a steady state is established after 6 days (Jeris 1983; Buffieare et al. 2000).

The AFBR thus seems to be capable of performing at relatively low temperatures with both low and high COD waste waters, without significant shock loading effects. Engineering improvements which can potentially minimize the mechanical power required for fluidization include reduction of the expanded volume, selection of a low-density medium of high specific area; and avoidance of fragility. Media such as sand, quartzite, alumina, anthracite, granular activated carbon, or cristobalite with a particle size of approximately 0.5 mm are usually employed (Buffieare et al. 2000).

#### Two-phase methane fermentation processes

Novel bioreactors for biogas fermentation such as the UASB, UAFP, and AFBR experience inherent problems when operated at high COD loads, due to the fact that the overall growth rate of acidogenic bacteria proceeds faster (tenfold) than that of methanogenic bacteria. When this occurs, inhibitory products such as volatile fatty acids and H_2_ accumulate in the reactor, slowing down the entire process. To overcome this, two-phase processes consisting of acidogenic and methanogenic fermentations have been investigated (Bowker 1983; Ke and Shi 2005; Xie et al. 2012; Sharma et al. 2012; Heeg et al. 2014; Berni et al. 2014) (Fig. 3d). In addition, since SS in waste water greatly influences the performance of the UASB or UAFP, an acidogenic fermentation first phase in combination with a UASB or UAFP second phase is useful in reducing the SS which enter the second phase.

In one full-scale two-phase system 70–97 % COD removal and biogas production of 3–13 kg/m^2^ day with a methane content of 65–80 % was obtained when operated at COD loads of 20–60 kg/m^3^/day for acidogenic fermentation (1st phase) and 6–30 kg/m^3^/day for methanogenic fermentation (2nd phase). In another example, a two-phase system consisting of a complete stirred reactor for the first phase and a UASB for the second phase was constructed. When this system was applied in the treatment of alcohol distillery waste (COD = 10,000 mg/l) at HRTs of 16–72 h in the first phase, and 14 h in the second phase, 84 % COD removal and 92 % BOD removal were accomplished. A two-phase system consisting of a UAFP for the first phase and a horizontal AFP for the second phase has also been proposed, with which it should be possible to treat sewage waste water (COD 800–2600 mg/l) at HRTs of 2–5.5 h with a high methane content (~90 %) (Berni et al. 2014).

## Conclusion

Worldwide energy consumption and demand are growing up since past 50 years. With the growth of population, demand for energy is also increasing leading to an uneven supply and distribution of resources. Therefore, the requirement of sustainable and eco-friendly energy in India to satisfy the energy demand is inevitable. Along with the source of sustainable green energy, biogas production is an alternative way to produce clean energy through solid waste management. As it is produced by the action of several microbes upon the waste products, knowledge about the eco-physiology of the microbes will help in understanding their particular roles. Bearing in mind that the higher biogas production rate of the thermophilic system must have been accompanied by intensified intermediate production, it is noteworthy that the concentration of VFA within the UAFP effluent was equally low at both temperatures. Consequently, the acetogenesis and methanogenesis steps must also have been more active and the intermediates from hydrolysis and acidogenesis were instantly converted to methane within the thermophilic UAFP reactor. For biogas production methanogenesis is often the rate-limiting step. However, when plant biomasses are used as substrate, hydrolysis is the rate-limiting step because of higher content of lignocellulosic materials. Thus, to enhance the overall production rate in such processes, it is necessary to understand the primary degradation steps, i.e., hydrolysis and acidogenesis, for the control and optimization of the whole process. As all the microbes involved in AD are not culturable, attempts could be made to design ideal media and optimize the growth conditions for the non-culturable microbes with the aid of metagenomic improvements, so that extensive research could be done in cost-effective and easier ways. The eco-physiological effect of a microbe in the consortium can also be understood properly only if it can be cultured in vitro. Several microbes detected in the AD system have been found to be methane oxidizers and sulphate reducers, which are hindrances to the yield of biogas. Thus, studies on inhibiting the growth of such microbes would be beneficial for the biogas yield. Besides, the performance of AD in terms of biogas production is dependent upon the activity of several ion-specific transporters and enzyme systems. Detailed information on structure and biosynthesis of all the enzymes, biogenesis of the prosthetic groups involved in such enzyme systems is also not readily available. Hence, further studies could be designed to explore these steps. Fishing for these high-efficiency genes that control these enzyme systems will ultimately increase the production of biogas and sustain the production plant.
